# Supplementary material for: Using the Behaviour Change Wheel to develop an intervention to improve conversations about recovery on the stroke unit
Source: PLoS One. 2025 Jan 7;20(1):e0317087. doi: 10.1371/journal.pone.0317087 (PMC11706387; doi:10.1371/journal.pone.0317087)
Supplement: S1 File — (PDF) [file pone.0317087.s001.pdf]

Supplementary File 1: Completed GUIDED checklist

GUIDED – a guideline for reporting for intervention development studies

Duncan E, O'Cathain A, Rousseau N*, et al*. Guidance for reporting intervention development studies in health research (GUIDED): an evidence-based consensus study *BMJ Open* 2020;**10:**e033516. doi: 10.1136/bmjopen-2019-033516

	Item description	Page in manuscript where item is located
1	Report the context for which the intervention was developed.	3-4
2	Report the purpose of the intervention development process.	3-4
3	Report the target population for the intervention development process.	3-5
4	Report how any published intervention development approach contributed to the development process	4-6
5	Report how evidence from different sources informed the intervention development process.	6-8, 9-12
6	Report how/if published theory informed the intervention development process.	4-9
7	Report any use of components from an existing intervention in the current intervention development process.	N/A
8	Report any guiding principles, people or factors that were prioritised when making decisions during the intervention development process.	4
9	Report how stakeholders contributed to the intervention development process.	9-12
10	Report how the intervention changed in content and format from the start of the intervention development process	12-24
11	Report any changes to interventions required or likely to be required for subgroups.	27-28
12	Report important uncertainties at the end of the intervention development process.	26-30
13	Follow TIDieR guidance when describing the developed intervention.	N/A – intervention still in development
14	Report the intervention development process in an open access format.	Planned
