# Supplementary material for: Using the Behaviour Change Wheel to develop an intervention to improve conversations about recovery on the stroke unit
Source: PLoS One. 2025 Jan 7;20(1):e0317087. doi: 10.1371/journal.pone.0317087 (PMC11706387; doi:10.1371/journal.pone.0317087)
Supplement: Attachment — Submitted filename: Response to Reviewers.docx [file pone.0317087.s002.docx]

**Response to Reviewers**

|  | **Editor/ reviewer comments** | **Author’s response** |
| --- | --- | --- |
|  | **Editor** |  |
| 1 | Please ensure that your manuscript meets PLOS ONE's style requirements, including those for file naming. The PLOS ONE style templates can be found at  https://journals.plos.org/plosone/s/file?id=wjVg/PLOSOne_formatting_sample_main_body.pdf and  https://journals.plos.org/plosone/s/file?id=ba62/PLOSOne_formatting_sample_title_authors_affiliations.pdf | Thank you for this feedback. We have used the guidance provided to ensure the manuscript meets the journal’s style requirements. |
| 2 | Thank you for stating the following financial disclosure: Funding for this work was provided by the Stroke Association, via its Postgraduate Fellowship programme [ref: TSA PGF 2017-02].    Please state what role the funders took in the study.  If the funders had no role, please state: ""The funders had no role in study design, data collection and analysis, decision to publish, or preparation of the manuscript.""  If this statement is not correct you must amend it as needed.  Please include this amended Role of Funder statement in your cover letter; we will change the online submission form on your behalf. | This is indeed the case; the funders had no role in study design, data collection and analysis, decision to publish, or preparation of the manuscript, we have included this statement within our cover letter. |
| 3 | We note that you have indicated that there are restrictions to data sharing for this study. For studies involving human research participant da  6347ta or other sensitive data, we encourage authors to share de-identified or anonymized data. However, when data cannot be publicly shared for ethical reasons, we allow authors to make their data sets available upon request. For information on unacceptable data access restrictions, please see http://journals.plos.org/plosone/s/data-availability#loc-unacceptable-data-access-restrictions.  Before we proceed with your manuscript, please address the following prompts:  a) If there are ethical or legal restrictions on sharing a de-identified data set, please explain them in detail (e.g., data contain potentially identifying or sensitive patient information, data are owned by a third-party organization, etc.) and who has imposed them (e.g., a Research Ethics Committee or Institutional Review Board, etc.). Please also provide contact information for a data access committee, ethics committee, or other institutional body to which data requests may be sent.  b) If there are no restrictions, please upload the minimal anonymized data set necessary to replicate your study findings to a stable, public repository and provide us with the relevant URLs, DOIs, or accession numbers. Please see http://www.bmj.com/content/340/bmj.c181.long for guidelines on how to de-identify and prepare clinical data for publication. For a list of recommended repositories, please see https://journals.plos.org/plosone/s/recommended-repositories. You also have the option of uploading the data as Supporting Information files, but we would recommend depositing data directly to a data repository if possible.  Please update your Data Availability statement in the submission form accordingly. | We are keen to share our data where possible. De-identified data from our survey study is available online within a data repository <https://osf.io/9vyxd>  (this link is provided within the manuscript). There are some restrictions on public access to data from the ethnographic study which informed this research. These restrictions relate to the consent provided by the participants in the study for access to data they provided for the study (approved by the Health Research Authority (HRA) for the United Kingdom (UK) and the Research Ethics Committee (REC) (Yorkshire & the Humber (Bradford-Leeds). We have revised our Data Availability Statement in the online submission system to provide more detail. |
| 4 | Please include your full ethics statement in the ‘Methods’ section of your manuscript file. In your statement, please include the full name of the IRB or ethics committee who approved or waived your study, as well as whether or not you obtained informed written or verbal consent. If consent was waived for your study, please include this information in your statement as well. | These details have been added to the manuscript (page 7, lines 182-183 (page numbers refer to the version of the manuscript with tracked changes)). |
| 5 | Your abstract cannot contain citations. Please only include citations in the body text of the manuscript, and ensure that they remain in ascending numerical order on first mention. | No citations are included in the abstract; however, there is a numbered list, which we acknowledge could cause confusion. We have amended this to make this clearer. |
| 6 | Please include captions for your Supporting Information files at the end of your manuscript, and update any in-text citations to match accordingly. Please see our Supporting Information guidelines for more information: http://journals.plos.org/plosone/s/supporting-information. | These details have been added at the end of the document and the in-texts citations have been checked and amended where necessary. |
| 7 | Overall, throughout the manuscript, the relationship between BCW, COM-B and TDF and how they were applied is not clear. A diagram illustrating this relationship might help show this more clearly. Presumably it was an iterative process? | We have worked through the manuscript to ensure consistency of terminology and added clear definitions. We have included a diagram (Fig 1) demonstrating the links between the COM-B model and the TDF domains. Fig 2 and Table 4 also show these links within the context of our developed intervention. We hope this aids the clarity of our reporting. |
| 8 | Abstract conclusion needs revising. It should more accurately reflect your findings rather than stating what you did. Perhaps phrasing it along the lines of "the application of the COM-B (or is it BCW?) to intervention development for......was successful......further research will...... The conclusion should match the conclusion in the main body of the manuscript. | Thank you for this feedback. We have revised the abstract conclusion (page 2) and ensured it matches with the conclusion in the manuscript (page 30-31). |
| 9 | Background The aim of the work needs stating. You may also consider stating some objectives which could link to the different phases of the research. This would help structure the methods and results. | Thank you for this suggestion. We have clarified our aim and added objectives (page 5), which we feel does improve the structure of the manuscript. |
| 10 | Methods There is a lack of consistency in your use of COM-B and BCW. COM-B model applied in first paragraph, but then in phase 1 it is BCW. Also in your use of terms such as multi-phase, then you mention phase 1. Be very clear how many phases there were in the first paragraph and briefly describe them before going into the details in subsequent paragraphs. | Thank you for pointing this out. We have reviewed the text throughout and amended to provide consistency in terminology. We have also amended the text to be clearer about the number of phases and briefly described them on pages 5-6. |
| 11 | There is scant information on how the TDF was applied - please expand on this. Why was it used? | We have added further detail about this on pages 7-8. |
| 12 | More detail is required on how APEASE was judged or applied? | The APEASE criteria were judged subjectively, in line with Michie et al.’s guidance, first by LB and then through discussion with the wider team. Further detail has been added to the manuscript to reflect this (page 8) |
| 13 | The rationale for only shortlisting 5/6 barriers is required. | We have added this detail (page 11). |
| 14 | How were the barriers mapped to the TDF domains? | We have added more detail about how the TDF was used within the methods section (pages 7-8). |
| 15 | 47 stakeholders in the text but 48 in the table. | Thank you for pointing out this typographical error; it has now been corrected. |
| 16 | You selected 6 barriers but the results indicated that all were very or extremely important to address. This needs further explanation and the implications of this need picking up in the discussion. | To clarify, the goal of this work was prioritisation of those felt most important. However, we recognise this is as a limitation and have added more detail about the potential implications of this to the discussion (page 29) |
| 17 | Table 4 describes intervention components but how these were specified and selected needs explanation. | The components described in Table 4 were specified and selected through the application of the BCW approach, and subsequently whittled down through professionals’ feedback via the survey (described in the methods section). We have provided more detail in the methods section, which we hope adds clarity to the process. |
| 18 | The initial paragraph should answer the extent to which you addressed your aim rather than re-stating your aim. The first sentence could be broken into 2 to make a clearer message. | Thank you for this feedback. This paragraph has been amended (page 29). |
| 19 | Please clarify if the selection of barriers was informed by the survey rather than previous work. Previous work identified them but you selected the ones to address in the survey? | This is correct, the text has been amended to make this clearer (page 25). |
| 20 | The discussion would benefit from re-structuring so that you clearly describe what you found first before you make comparisons with the wider literature. | The discussion has been restructured, with the first paragraph focusing on findings (page 25). |
| 21 | Line 395 - rather than describing the MRC framework and BCW again discuss how these facilitated your research aims and objectives. Discuss the application of it rather than rationale for it's use. | Thank you for this feedback; we have focussed the discussion on the application of the MRC framework and BCW approach (pages 25-26). |
| 22 | Expand on your statement that the final intervention will be flexible - what does this mean and how will it be adaptable to other settings, populations etc. | We have added more detail to this paragraph and provided examples (see pages 27-28). |
| 23 | line 380-382 - this belongs in the introduction/background section as it provides a rationale for your study. | These lines have been moved into the introduction section. |
| 24 | Conclusion This should align with the abstract conclusion and needs improving. You used frameworks and processes for intervention development. To what extent did these enable you to achieve your aims, any limitations and what comes next. | We have rewritten our conclusion and ensured it corresponds with the conclusion in our abstract (pages 30-31). |
|  | **Reviewer 1** |  |
| 25 | This is an interesting manuscript and although I am from a different discipline, it was exciting to consider how this approach could be applied across different areas of healthcare. Please find below a couple of minor comments to consider which could be clarified further to enhance the readability / improve the accessibility and add to the interest of the paper. Some readers may be reading your paper for your methodological approach rather than topic itself, so it is good to consider this too. | We thank the reviewer for their kind comments and have considered how readers may be concerned with the methodological approach as we have revised our manuscript; our aim is to make this as clear as possible as an exemplar. We have provided more detail to this end, and in line with the editor’s comments. |
| 26 | Maybe consider providing a little more detail about the TDF. This would help to show the reader why you used both the TDF and COM-B, rather than one or the other. | We agree that more information would be helpful for readers and note that the editor has made a similar comment (point 11). We have added more detail to the methods section to address this (pages 7-8). |
| 27 | Overall, I felt that you have explained quiet a complicated methodology well and the tables do help to clarify the different aspects and show your rational and explanations for each. It did take some time to understand and follow all of the stages, so if there was a way to reduce / make any sections more concise, this would help the overall readability, but I understand that this may not be possible to do without losing the required level of detail. | We agree with the reviewer that the methodology is challenging to describe clearly and yet succinctly. We hope that our revisions add clarity and that the detail and rationale for our decision-making helps fellow intervention developers. |
